# Efficacy and safety of rTMS for global symptom severity, negative symptoms, and auditory hallucinations in treatment-resistant schizophrenia: a systematic review and meta-analysis

**DOI:** 10.3389/fpsyt.2026.1783758

**Published:** 2026-07-24

**Authors:** Mohammed A. Alhassan, Mohammed A. Alarabi, Waled M. Albalawi, Thenuwara Arachchige Omila Kasun Meetiyagoda, Anum Nisar, John Robert M. Torres

**Affiliations:** 1 Division of Psychiatry, Department of Medical Specialties, College of Medicine, Majmaah University, Al Majmaah, Saudi Arabia; 2 Department of Psychiatry, College of Medicine, King Saud University, Riyadh, Saudi Arabia; 3 Department of Psychiatry, King Salman Armed Forces Hospital, Tabuk, Saudi Arabia; 4 Saitama University, Saitama, Japan; 5 Institute of Population Health, University of Liverpool, Liverpool, United Kingdom; 6 Independent Researcher, Laguna, Philippines

**Keywords:** efficacy, safety, rTMS (repetitive transcranial magnetic stimulation), schizophrenia, treatment-resistant schizophrenia (TRS)

## Abstract

**Background:**

Treatment-resistant schizophrenia (TRS) affects up to one-third of individuals with schizophrenia and remains a major clinical challenge despite optimized pharmacotherapy. Repetitive transcranial magnetic stimulation (rTMS) has been proposed as a neuro-modulatory intervention for refractory symptoms, yet evidence specific to TRS remains limited and inconsistent.

**Objectives:**

To evaluate the efficacy and safety of rTMS for reducing global symptom severity, negative symptoms, and auditory hallucinations in adults with treatment-resistant schizophrenia, and to examine the influence of stimulation parameters on treatment outcomes.

**Methods:**

A systematic review and meta-analysis was conducted following PRISMA guidelines and registered with PROSPERO (CRD420251143558). Eight electronic databases were searched up to 10^th^ September 2025. Randomized controlled trials including adults with TRS and comparing rTMS with sham stimulation were eligible. Standardized mean differences (Hedges’ g) were pooled using REML-based random-effects models. Heterogeneity and subgroup effects were examined, and publication bias was explored through visual inspection of funnel plots.

**Results:**

Across included trials, rTMS was not associated with statistically significant improvements in overall symptom severity measured by PANSS (SMD 0.14, 95% CI −0.26 to 0.54), negative symptoms (SMD 0.37, 95% CI −0.12 to 0.86), or auditory hallucinations measured by AHRS (SMD −0.03, 95% CI −0.26 to 0.20), based on post-treatment outcome scores. Substantial heterogeneity was observed for PANSS and negative symptoms, while heterogeneity for AHRS was low. Subgroup findings were exploratory and should be interpreted cautiously.

**Conclusions:**

Current evidence does not support a consistent therapeutic benefit of rTMS over sham stimulation for symptom reduction in treatment-resistant schizophrenia. While some subgroup analyses suggested variability in effect estimates across stimulation protocols, these findings were exploratory and should be interpreted with caution. Further large-scale, well-designed trials are needed to clarify the role of rTMS in this population.

**Systematic review registration:**

https://www.crd.york.ac.uk/PROSPERO/view/CRD420251143558, identifier CRD420251143558.

## 1 Introduction

Schizophrenia is a chronic and disabling mental disorder characterised by positive symptoms, such as hallucinations and delusions, negative symptoms, including affective blunting and reduced motivation, and significant cognitive and functional impairments (1). Despite advances in antipsychotic pharmacotherapy, a substantial proportion of individuals fail to achieve adequate symptom control (2, 3). It is estimated that approximately 20–30% of patients develop treatment-resistant schizophrenia (TRS), typically defined by lack of response to at least two adequate antipsychotic trials (4–7). TRS is associated with persistent symptom burden, impaired quality of life, increased rates of hospitalisation, elevated suicide risk, and substantial healthcare costs (4).

Clozapine remains the most effective pharmacological treatment for TRS and is recommended as the gold-standard intervention (8). However, its clinical utility is limited by incomplete response in a significant proportion of patients, poor tolerability, and the need for intensive monitoring due to potentially serious adverse effects (9, 10). As a result, many individuals with TRS continue to experience refractory positive and negative symptoms despite optimal pharmacological management (11). This therapeutic gap has driven increasing interest in adjunctive non-pharmacological approaches aimed at modulating the neurobiological mechanisms underlying symptom persistence.

Repetitive transcranial magnetic stimulation (rTMS) is a non-invasive neuromodulation technique that delivers focal magnetic pulses to targeted cortical regions, inducing changes in neuronal excitability and connectivity (12). rTMS has demonstrated efficacy and safety in several psychiatric conditions, most notably major depressive disorder, and is supported by evidence-based clinical recommendations for therapeutic use in this population (13). In schizophrenia, rTMS has been investigated for its potential to reduce auditory hallucinations, typically via low-frequency stimulation of the temporoparietal cortex (often left-sided), and to ameliorate negative symptoms through high-frequency stimulation of the dorsolateral prefrontal cortex (commonly left-sided). However, stimulation parameters vary considerably across studies, including differences in frequency (1–20 Hz), intensity (100–120% motor threshold), coil type (e.g., H-coil), treatment duration, and cortical targets (14). Proposed mechanisms underlying the efficacy of rTMS in schizophrenia include modulation of dysfunctional cortical-subcortical circuits, regulation of aberrant dopaminergic activity, and enhancement of neuroplasticity (15).

While a growing body of research has examined the effects of rTMS in schizophrenia, findings remain inconsistent (8, 16). This heterogeneity is particularly pronounced in studies involving individuals with TRS, reflecting variability in diagnostic definitions, stimulation parameters (including frequency, intensity, cortical target, and treatment duration), outcome measures, and concomitant pharmacological treatments. More importantly, existing systematic reviews often combine treatment-responsive and treatment-resistant patients, limiting the clinical applicability of their conclusions to TRS patients, which represent a distinct subgroup within the larger heterogeneous group of individuals with schizophrenia.

To date, the evidence for rTMS in TRS has not been comprehensively synthesised in a manner that focuses explicitly on this clinically distinct subgroup. Given the ongoing need for effective adjunctive treatment options in TRS, a rigorous evaluation of both efficacy and safety is essential to inform clinical decision-making and future research directions. Therefore, the present systematic review and meta-analysis aims to evaluate the efficacy and safety of rTMS in adults with TRS. Specifically, this review assessed the impact of rTMS on global symptom severity, negative symptoms, and auditory hallucinations in adults with treatment-resistant schizophrenia, while also examining adverse events, treatment discontinuation, and differences across stimulation protocols. By consolidating the current evidence base, this study seeks to address an important gap in the literature and contribute to the optimisation of treatment strategies for individuals with TRS.

## 2 Materials and methods

This systematic review and meta-analysis was registered in the International Prospective Register of Systematic Reviews (PROSPERO; CRD420251143558) and is publicly accessible. The registered protocol specified that databases would be searched for articles published from 1 January 2010 onward, with no restriction on the search end date. During the conduct of the review, the search was broadened to include all records from database inception to 10 September 2025 to increase comprehensiveness and reduce the risk of missing earlier eligible randomized controlled trials. No other deviations from the registered protocol occurred. The review was conducted in accordance with the Preferred Reporting Items for Systematic Reviews and Meta-Analyses (PRISMA) guidelines. The completed PRISMA 2020 checklist is provided in the **Supplementary Material**.

### 2.1 Search strategy

A comprehensive literature search was conducted across MEDLINE, Embase, and PsycINFO via OVID, PubMed, Scopus, Web of Science, ClinicalTrials.gov, and the Cochrane Library. All databases were searched from inception to 10^th^ September 2025. The search strategy combined terms related to TRS and rTMS. Reference lists of eligible studies were also screened to identify additional articles. Detailed search strategy is given in **Supplementary Materials** **Table 1**.

### 2.2 Inclusion criteria

- Randomized controlled trials (RCTs), including parallel-group and randomized cross-over designs (first-phase data only)
- Adults aged 18 to 65 years
- Diagnosis of treatment-resistant schizophrenia or persistent refractory schizophrenia symptoms consistent with treatment resistance.
- Evaluation of rTMS as monotherapy or adjunctive treatment
- Comparison with sham or placebo stimulation

Only studies published in peer-reviewed journals and available in English were considered eligible.

### 2.3 Exclusion criteria

• Studies of non-TRS
• Psychotic disorders other than schizophrenia
• Non-randomized or uncontrolled designs

### 2.4 Study selection

Study selection was conducted independently by two reviewers (T.A.O.K.M. and J.T.) in two stages. First, titles and abstracts were screened to identify potentially relevant studies. Full-text articles were then retrieved and assessed using the predefined eligibility criteria. Any discrepancies between reviewers were resolved through discussion or, when necessary, consultation with a third reviewer (M.A.Alh). Final study inclusion was determined by consensus.

### 2.5 Data extraction

Data extraction was performed independently by two reviewers using a standardized extraction form. For each study, information was collected on study characteristics (including author, year, country, and design), sample size and participant demographics, and definitions of treatment resistance.

TRS was defined as failure to respond to at least two adequate antipsychotic trials, in line with established clinical criteria. To ensure consistency, included studies were re-examined at the full-text level to verify TRS definitions. Studies were classified into the following categories: (i) TRS (≥2 failed antipsychotic trials), (ii) clozapine-resistant TRS, (iii) TRS (author-defined), and (iv) TRS not specified, where explicit criteria were not reported. Studies without explicit TRS definitions were retained if they included patients with persistent symptoms (e.g., auditory hallucinations or predominant negative symptoms) and were considered in interpretation.

Detailed rTMS protocol parameters such as stimulation target, frequency, intensity, and number of sessions were also extracted.

Outcome measures were extracted and categorised into predefined domains based on validated symptom scales. Global symptom severity was assessed using the Positive and Negative Syndrome Scale (PANSS total), negative symptoms were assessed using either the Scale for the Assessment of Negative Symptoms (SANS) or the PANSS-negative subscale, and auditory hallucinations were assessed using the Auditory Hallucination Rating Scale (AHRS). Where multiple measures were reported for the same domain within a study, only one outcome was included to avoid duplication.

Secondary outcomes included adverse events, treatment discontinuation, and other safety-related indicators.

Negative symptom outcomes included both SANS and PANSS-negative subscale measures, which were combined into a single domain using standardized mean differences (SMDs).

Where outcome data were incomplete or reported in a format that did not permit extraction of post-treatment means and standard deviations, studies were excluded from the quantitative synthesis and retained in the qualitative review where appropriate. Study authors were not contacted to obtain additional data, and no statistical imputation of missing outcome values or summary statistics was performed. Only studies providing sufficient data for effect size calculation were included in the meta-analysis.

### 2.6 Risk of bias assessment

Risk of bias was assessed independently by two reviewers using the Cochrane Risk of Bias 2 (RoB 2) tool at the outcome level. The following domains were evaluated: (1) bias arising from the randomization process, (2) deviations from intended interventions, (3) missing outcome data, (4) measurement of the outcome, and (5) selection of the reported result. Each outcome was judged as low risk, some concerns, or high risk of bias, with disagreements resolved through discussion.

### 2.7 Effect size calculation

For each scale, post-treatment means and standard deviations were extracted for active and sham groups. SMDs (Hedges’ g) were calculated using post-treatment outcome scores to compare active and sham stimulation groups at the end of treatment. This approach was considered appropriate because all included studies were randomized controlled trials, in which randomization is expected to achieve reasonable baseline comparability between treatment groups. Baseline symptom scores for the active and sham groups were extracted and are presented in **Supplementary Table 2**, allowing assessment of baseline comparability across included studies.

### 2.8 Statistical analysis

Meta-analyses were conducted in R (version 4.5.0) using the *meta* package. Continuous outcomes were synthesized using SMDs (Hedges’ g) computed from post-treatment means and standard deviations reported for the active and sham groups. All analyses used the inverse-variance method and a random-effects framework to account for anticipated methodological and clinical heterogeneity. Between-study variance (τ²) was estimated using restricted maximum likelihood (REML), and 95% confidence intervals for τ² and τ were obtained using the Q-profile method. Heterogeneity was assessed using Cochran’s Q statistic and the I² statistic.

Fixed-effect models were initially examined for comparison; however, given the anticipated clinical and methodological heterogeneity across trials, including differences in patient populations, stimulation targets, frequencies, treatment duration, and outcome measures, random-effects models were used throughout. This approach was further supported by the observed heterogeneity in the main analyses, which was substantial for PANSS total and negative symptom outcomes. To assess the robustness of the findings, leave-one-out sensitivity analyses were performed for all primary outcomes. In addition, Hartung-Knapp adjusted confidence intervals were calculated as a sensitivity analysis to evaluate the impact of small-sample adjustment on the pooled estimates.

For trials with multiple active treatment arms or multiple comparisons against a shared sham/control group, we applied standard methods to avoid unit-of-analysis errors. Specifically, when multiple active arms were compared with a single sham group, the sham group sample size was proportionally divided across comparisons to prevent double-counting. Where appropriate, multiple active arms with similar stimulation parameters were combined into a single group. Each study contributed only one independent effect size per outcome to the primary meta-analysis.

Cross-over trials were included; however, to avoid potential carryover effects, only first-phase data were used in the meta-analysis. Where first-phase data were not extractable, studies were included in the qualitative synthesis but excluded from quantitative analysis.

For ease of interpretation, sham stimulation was coded as the reference group and active rTMS as the comparator group in all analyses. This coding convention was adopted so that positive SMD values consistently indicate outcomes favouring active rTMS, whereas negative values indicate outcomes favouring sham stimulation, thereby improving readability and interpretation of the forest plots.

### 2.9 Subgroup analyses

Stimulation protocols were categorised based on frequency and cortical target to enable subgroup analyses. Low-frequency stimulation was defined as <5 Hz, high-frequency stimulation as ≥5 Hz, and intermittent theta burst stimulation (iTBS) was considered a separate category because of its distinct patterned mode of delivery. Studies applying multiple stimulation frequencies were classified as mixed protocols. Stimulation targets were grouped into dorsolateral prefrontal cortex (DLPFC), temporoparietal cortex, temporal cortex, and cerebellar regions.

Subgroup analyses were conducted to explore whether these stimulation parameters influenced treatment outcomes. Where sufficient data were available, analyses were stratified by stimulation frequency and cortical target.

Due to variability in reporting of stimulation intensity (e.g., percentage of motor threshold) and total pulse dose, these parameters were extracted but were not consistently included in subgroup analyses.

### 2.10 Certainty of evidence

A formal certainty-of-evidence assessment using the Grading of Recommendations, Assessment, Development, and Evaluations (GRADE) framework was not conducted. The primary objective of this review was to evaluate the efficacy and safety of rTMS in treatment-resistant schizophrenia through quantitative synthesis of randomized controlled trials. Interpretation of the findings was informed by outcome-level risk-of-bias assessments, heterogeneity estimates, sensitivity analyses, prediction intervals, and subgroup analyses. Given the relatively small number of studies available for several outcomes, substantial clinical and methodological heterogeneity across stimulation protocols, and the exploratory nature of several analyses, a formal GRADE assessment was not undertaken.

## 3 Results

### 3.1 Study selection

**Figure 1:**
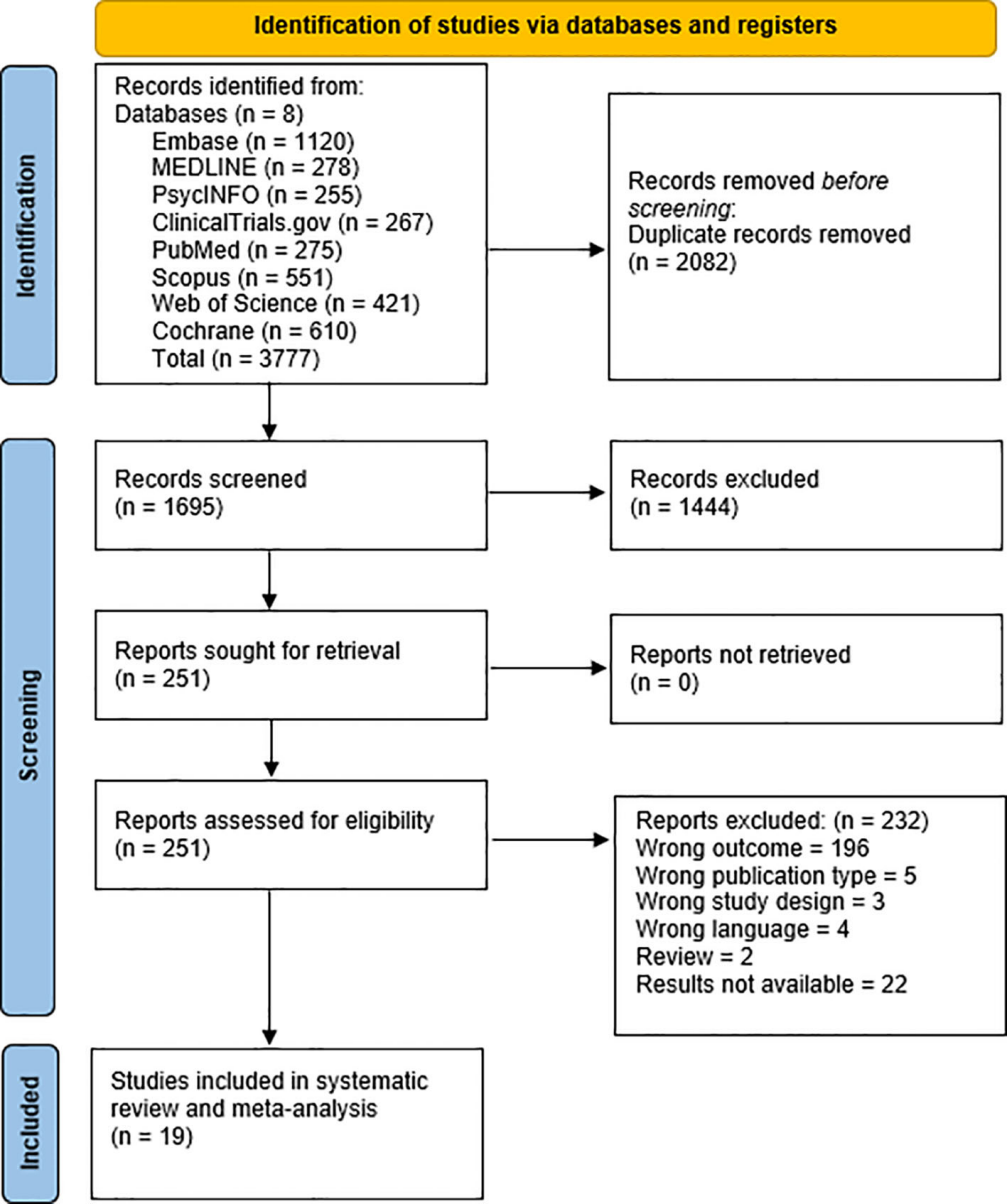
PRISMA flow diagram.

The database search and reference screening yielded 1,695 unique records, of which 251 reports were retained for full-text review (**Figure 1**). After applying the inclusion and exclusion criteria, a total of 19 RCTs were included in the meta-analysis (7, 17–34). The characteristics of the included studies are summarized in **Table 1**. Details of the study selection process are presented in the PRISMA diagram. Of the 19 included RCTs, 3 crossover trials (24, 28, 29) were excluded from quantitative synthesis due to unavailable first-phase data, and one study (27) lacked outcome-level data for RoB assessment, resulting in 15 studies included in the risk-of-bias evaluation.

**Table 1:** Characteristics of included randomized controlled trials evaluating repetitive transcranial magnetic stimulation (rTMS) for global symptom severity, negative symptoms, and auditory hallucinations in treatment-resistant schizophrenia.

Sr	Author, year	Country	Design	Sample size (active/sham)	Age, mean ± SD (active/sham)	TRS definition/population criteria	Target site	Frequency	Sessions	Outcomes
1	Bais, 2014	Netherlands	RCT	31/16	37.2 ± 14.9/37.3 ± 11.6	≥2 failed antipsychotic trials	Temporoparietal cortex	LF	12	AHRS; PANSS (Total); PANSS-Negative
2	Basavaraju, 2021	India	RCT	30/30	31.5 ± 7.3/30.7 ± 7.0	Predominant negative symptoms (TRS not specified)	Cerebellum	iTBS	10	SANS
3	Blumberger, 2012	Canada	RCT	34/17	36.6 ± 8.2/40.8 ± 12.1	Auditory hallucinations (TRS not specified)	Temporoparietal cortex	LF (priming protocol)	20	AHRS; PANSS (Total)
4	Chauhan, 2021	India	RCT	19/17	41.7 ± 8.9/39.4 ± 8.2	TRS (as defined by authors)	Cerebellum	iTBS	10	PANSS (Total); PANSS-Negative
5	de Jesus, 2011	Brazil	RCT	8/9	46.0 ± 9.8/36.5 ± 6.4	Clozapine-treated TRS	Temporoparietal cortex	LF	20	AHRS
6	Du, 2022	China	RCT	25/22	45.9 ± 10.0/45.1 ± 10.4	Chronic schizophrenia (TRS not specified)	DLPFC	HF	20	PANSS (Total); SANS
7	Fitzgerald, 2008	Australia	RCT	12/8	37.2 ± 10.4/33.2 ± 9.8	≥2 failed antipsychotic trials	DLPFC	HF	15	PANSS (Total); SANS
8	Kim, 2014	Korea	RCT (cross-over)	22/22	36.6 ± 9.5 (Mixed)	Auditory hallucinations (TRS not specified)	Temporoparietal cortex/DLPFC	Mixed (LF + HF)	6–10	AHRS
9	Kimura, 2016	Japan	RCT	16/14	44.6 ± 10.5/40.7 ± 9.0	Auditory hallucinations (TRS not specified)	Temporoparietal cortex	HF	4	AHRS
10	Koops, 2016	Netherlands	RCT	37/34	38.0 ± 15.0/42.0 ± 13.0	Auditory hallucinations (TRS not specified)	Temporoparietal cortex	HF	10	AHRS
11	Lee, 2005	Korea	RCT	25/14	41.3 ± 10.3/40.7 ± 9.0	Auditory hallucinations (TRS not specified)	Temporoparietal cortex	LF	10	AHRS
12	Li, 2025	China	RCT	60/30	44.6 ± 9.4/39.9 ± 8.9	Chronic schizophrenia (TRS not specified)	DLPFC	Mixed (LF + HF)	20	PANSS (Total); PANSS-Negative
13	Loo, 2010	Australia	RCT (cross-over)	18/18	33.8 ± 12.2 (Mixed)	Auditory hallucinations (TRS not specified)	Temporal cortex	LF	30	AHRS
14	McIntosh, 2004	UK	RCT (cross-over)	16/16	35.9 ± 10.6 (Mixed)	Auditory hallucinations (TRS not specified)	Temporoparietal cortex	LF	8	PANSS-Negative
15	Paillère-Martinot, 2017	France	RCT	15/12	32.1 ± 6.8/31.3 ± 7.8	Auditory hallucinations (TRS not specified)	Temporoparietal cortex	LF	10	AHRS; SANS
16	Prikryl, 2007	Czech Republic	RCT	11/11	31.4 ± 8.4/36.5 ± 10.7	TRS (as defined by authors)	DLPFC	HF	15	PANSS (Total); PANSS-Negative
17	Singh, 2020	India	RCT	15/15	33.3 ± 9.8/29.8 ± 5.7	TRS (as defined by authors)	DLPFC	HF	20	SANS
18	Slotema, 2011	Netherlands	RCT	42/20	36.0 ± 10.0/41 ± 10.3	Auditory hallucinations (TRS not specified)	Temporal cortex	LF	15	AHRS
19	Wang, 2022	China	RCT	33/26	22.8 ± 2.5/24.2 ± 4.6	TRS (as defined by authors)	DLPFC	iTBS	42	PANSS (Total); SANS

TRS, treatment-resistant schizophrenia; DLPFC, dorsolateral prefrontal cortex; LF, low-frequency stimulation; HF, high-frequency stimulation; iTBS, intermittent theta burst stimulation; AHRS, Auditory Hallucination Rating Scale; PANSS, Positive and Negative Syndrome Scale; SANS, Scale for the Assessment of Negative Symptoms; RCT, randomized controlled trial; AVH, auditory verbal hallucinations; NR, not reported.

TRS classification was verified through full-text review. Studies were categorised as: TRS (≥2 failed antipsychotic trials), clozapine-resistant TRS, TRS (author-defined), or TRS not specified where explicit criteria were not reported.

Studies without explicit TRS criteria were retained if participants exhibited persistent symptoms consistent with treatment resistance.

Stimulation frequency categories were defined as low-frequency (<5 Hz), high-frequency (≥5 Hz), intermittent theta burst stimulation (iTBS), and mixed protocols where multiple frequencies were applied.

For cross-over trials, only first-phase data were considered for meta-analysis to avoid carryover effects.

### 3.2 Effects of rTMS on total symptom severity (PANSS total score)

**Figure 2:**
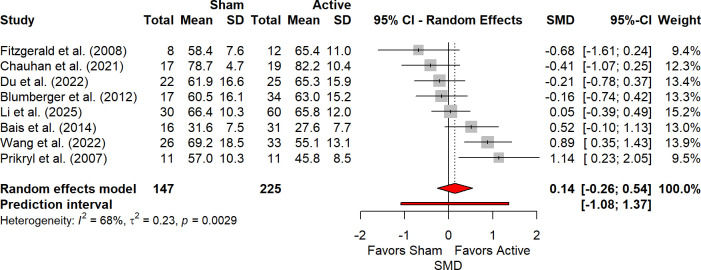
Forest plot of the standardized mean difference (SMD) in post-treatment global symptom severity (PANSS total scores) comparing active rTMS with sham stimulation across included studies.

A total of eight studies (7, 17, 19, 20, 22, 23, 31, 34) assessed overall symptom severity using the PANSS, comprising 372 participants (active n = 225; sham n = 147). The pooled SMD was 0.14 (95% CI −0.26 to 0.54), indicating no statistically significant difference between rTMS and sham stimulation. Individual study results displayed considerable variability, with effect sizes ranging from −0.68 (favouring sham) to 1.14 (favouring active). Substantial heterogeneity was observed (I² = 68%), suggesting variability in treatment effects across studies, likely reflecting differences in study design, patient populations, and stimulation protocols. A forest plot of PANSS outcomes is presented in **Figure 2**.

### 3.3 Effects of rTMS on negative symptoms (SANS and PANSS-negative subscale scores)

**Figure 3:**
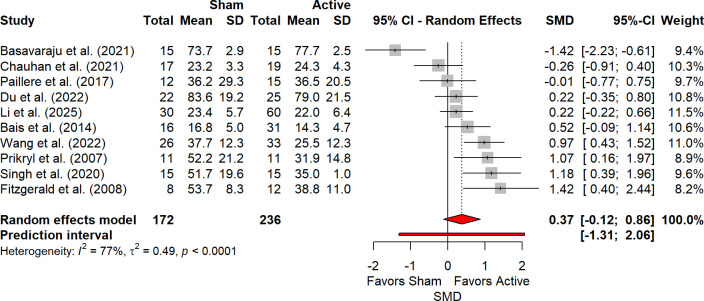
Forest plot of the standardized mean difference (SMD) in post-treatment negative symptom severity (SANS/PANSS negative subscale Scores) comparing active rTMS with sham stimulation across included studies.

Ten (7, 17, 18, 20, 22, 23, 30–32, 34) studies assessed negative symptoms using either the SANS or the PANSS-negative subscale. Across these studies, rTMS was not associated with a statistically significant improvement in negative symptoms compared with sham stimulation. The pooled SMD was 0.37 (95% CI −0.12 to 0.86), indicating no statistically significant difference between rTMS and sham stimulation. Substantial heterogeneity was observed (I² = 77%), reflecting considerable variability in treatment effects across studies. Individual study estimates ranged from −1.42 (favouring sham) to 1.42 (favouring active). Negative symptom outcomes included both SANS and PANSS-negative subscale measures, which were combined for analysis. A forest plot of negative symptom outcomes is shown in **Figure 3**.

### 3.4 Effects of rTMS on auditory hallucinations (AHRS Scores)

**Figure 4:**
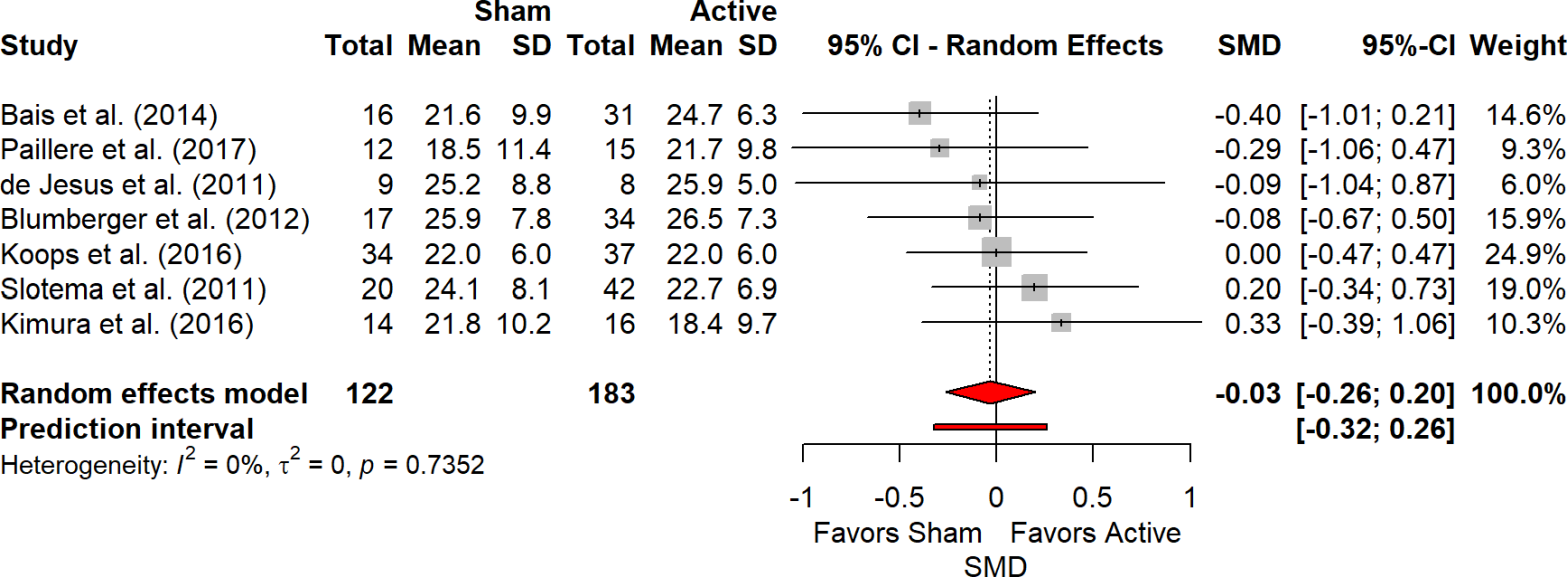
Forest plot of the standardized mean difference (SMD) in post-treatment auditory hallucination severity (AHRS Scores) comparing active rTMS with sham stimulation across included studies.

Seven studies (17, 19, 21, 25, 26, 30, 33) assessed auditory hallucinations using the AHRS, comprising a total of 305 participants (active n = 183; sham n = 122). The pooled effect of rTMS on auditory hallucinations was small and did not reach statistical significance (SMD −0.03, 95% CI −0.26 to 0.20). Effect estimates were distributed around the null, with individual study effects ranging from −0.40 (favouring sham) to 0.33 (favouring active). No statistical heterogeneity was detected (I² = 0%); however, this should be interpreted with caution given the small number of included studies and potential clinical and methodological heterogeneity across trials. A forest plot of AHRS outcomes is presented in **Figure 4**.

### 3.5 Subgroup analysis

Subgroup analyses were conducted to explore the potential influence of clinical and methodological factors, including stimulation site, patient characteristics, and study setting. Across outcomes, subgroup analyses largely mirrored the primary findings, with most comparisons remaining non-significant (**Supplementary Tables 4–6**).

Although some subgroups demonstrated statistically significant pooled effects, including prefrontal stimulation (SMD = 0.75, 95% CI [0.33, 1.17]) and studies focusing on patients with prominent negative symptoms (SMD = 0.76, 95% CI [0.11, 1.41]), these findings were based on small numbers of studies and should be interpreted cautiously. Formal tests of subgroup differences were not conducted because of limited statistical power, and therefore these results should be considered exploratory rather than definitive evidence of effect modification. Overall, while some subgroup-specific signals were observed, there was insufficient evidence to conclude that treatment effects differed reliably across stimulation protocols or patient subgroups.

### 3.6 Sensitivity analysis

Sensitivity analyses using a leave-one-out approach demonstrated that the overall findings were robust across all outcomes. For AHRS and PANSS total scores, omission of individual studies did not materially alter the pooled estimates or statistical significance. For negative symptoms, results remained non-significant across most iterations; however, exclusion of individual influential studies resulted in modest shifts in effect size and heterogeneity, suggesting that findings for this outcome are more sensitive to individual study effects. (**Supplementary Figures 1–3**).

Hartung-Knapp sensitivity analyses yielded results consistent with the primary random-effects models. The pooled effect estimates and overall conclusions remained unchanged for AHRS, PANSS total, and negative symptom outcomes, indicating that the findings were robust to small-sample adjustment of confidence intervals (**Figure 4**).

### 3.7 Qualitative synthesis of studies not included in meta-analysis

Three randomized crossover trials (24, 28, 29), were excluded from the quantitative synthesis due to unavailability of extractable first-phase data or incompatible reporting formats, and are summarised narratively here. Collectively, these studies enrolled 56 participants and assessed auditory hallucinations as the primary outcome using the AHRS, with two additionally reporting PANSS subscale data.

McIntosh et al. (29) found no significant difference between 1 Hz left temporoparietal rTMS and sham across hallucination and PANSS outcomes in 16 treatment-resistant patients, with both conditions producing comparable improvement from baseline. A trend toward greater benefit in the second treatment period regardless of condition suggested period effects may have obscured any true treatment signal. Loo et al. (28) similarly found no significant advantage for left or right temporal 1 Hz rTMS over vertex sham across 18 participants over three treatment days, though a trend toward reduced hallucination-related distress with active stimulation was noted. Modest improvements emerged during a subsequent open-label continuation phase, but without a sham comparator these cannot be attributed to specific rTMS effects. Kim et al. (24) compared four conditions, low-frequency and high-frequency bilateral temporoparietal rTMS, high-frequency bilateral Broca’s area rTMS, and sham in 22 patients, finding significant within-condition reductions in AHRS scores across all four modules including sham, with no significant treatment effects or time-by-treatment interactions.

Across all three studies, active rTMS did not demonstrate superiority over sham, and improvements in both conditions were consistently observed, likely reflecting placebo response or period effects. These qualitative findings are broadly consistent with the meta-analytic AHRS result (SMD −0.03, 95% CI −0.26 to 0.20), reinforcing the overall conclusion that current evidence does not support a reliable therapeutic benefit of rTMS for auditory hallucinations in treatment-resistant schizophrenia. These findings highlight the methodological limitations of crossover designs in rTMS trials, particularly the susceptibility to carryover and placebo effects, which may obscure true treatment signals.

### 3.8 Safety and tolerability

Safety outcomes were reported in 14 of the 19 included trials, although the extent and format of reporting varied considerably, precluding quantitative synthesis. Overall, rTMS was generally well tolerated, with most reported adverse events being mild, transient, and self-limiting.

The most commonly reported adverse events included headache, facial muscle twitching, scalp or stimulation-site discomfort, dizziness, light-headedness, nausea, fatigue, and transient sensory symptoms. For example, Bais et al. (17) reported transient headache, facial muscle twitching, light-headedness, earache, and tingling sensations, all of which resolved quickly without intervention. Similarly, Slotema et al. (33) reported mild adverse events including facial muscle twitching, headache, scalp discomfort, cervical pain, nausea, dizziness, abdominal pain, and fatigue, while Blumberger et al. (19) reported recurrent headaches and jaw or facial contraction discomfort. Mild headaches were also reported in studies by Chauhan et al. (20), de Jesus et al. (21), Fitzgerald et al. (23), and Lee et al. (27).

Serious adverse events were uncommon. Basavaraju et al. reported two cases of symptoms suggestive of mania or hypomania in participants receiving active cerebellar iTBS, whereas no such events occurred in the sham group. Singh et al. reported a seizure episode in one participant receiving active rTMS during the sixth treatment session, which resolved spontaneously and was followed by transient postictal drowsiness. Apart from these events, most studies explicitly reported no serious treatment-related adverse events, seizures, or clinically significant complications.

Treatment discontinuation due to adverse events was rarely reported. Several studies specifically noted that adverse events did not result in withdrawal from treatment, and affected participants were generally able to complete the intervention protocol. Overall, the available evidence suggests that rTMS is generally well tolerated in individuals with treatment-resistant schizophrenia. However, inconsistent adverse-event reporting and limited information regarding discontinuation restrict definitive conclusions regarding the comparative safety of different stimulation protocols.

### 3.9 Risk of bias

A total of 15 of the 19 included studies were assessed using the Cochrane Risk of Bias 2 tool. The remaining four studies were not assessed: three were crossover trials included only in the qualitative synthesis for which first-phase data were not extractable (24, 28, 29), and one study (27) did not report AHRS total scores in a format compatible with outcome-level domain appraisal. Among the 15 assessed studies, 8 were rated as low risk of bias, 6 as having some concerns, and 1 as high risk. Ratings of “some concerns” were primarily driven by incomplete reporting of allocation concealment and missing outcome data, while measurement of outcomes was generally considered low risk due to the use of standardized scales. (**Supplementary Table 3**).

### 3.10 Publication bias

Publication bias was explored through visual inspection of funnel plots (**Supplementary Figures 5-7**). Given the small number of studies available for each outcome (PANSS total: k = 8; negative symptoms: k = 10; AHRS: k = 7), formal statistical tests for funnel plot asymmetry and trim-and-fill analyses were not performed because these methods have limited reliability and interpretability in small meta-analyses. Visual inspection did not suggest marked asymmetry; however, publication bias and small-study effects cannot be excluded.

## 4 Discussion

This systematic review and meta-analysis synthesised evidence from randomized controlled trials evaluating rTMS in adults with TRS. Overall, rTMS did not demonstrate a statistically significant effect compared to sham stimulation across any of the evaluated domains, including auditory hallucinations, global symptom severity, and negative symptoms. While point estimates for some outcomes suggested a trend favouring active treatment, confidence intervals consistently crossed the null, and prediction intervals were wide for PANSS and negative symptom outcomes, indicating substantial uncertainty in expected effects across settings.

These findings suggest that, based on current evidence, rTMS does not provide a consistent or reliable therapeutic benefit for symptom reduction in TRS. Although earlier meta-analyses in broader schizophrenia populations have reported modest improvements, particularly for negative symptoms and auditory hallucinations (8, 16, 35), the present analysis focused specifically on treatment-resistant populations and does not confirm these effects. The absence of a similar effect in the present analysis may reflect the greater biological and clinical complexity of TRS, where neural circuits may be less responsive to modulation through non-invasive stimulation (36).

Substantial heterogeneity was observed, particularly for PANSS and negative symptom outcomes, indicating that variability in study findings is likely driven by differences in patient populations, stimulation parameters, and methodological factors. rTMS protocols varied substantially across studies, including differences in stimulation target, frequency, intensity, total pulses, and number of sessions, which may have contributed to heterogeneity and limits comparability across trials. Although subgroup analyses suggested variability in effect estimates across stimulation targets and protocols, these findings should be interpreted with caution, as no formal test of subgroup differences was conducted and observed differences may reflect chance or residual confounding.

From a mechanistic perspective, the lack of consistent benefit across outcomes highlights the challenge of applying rTMS as a broadly effective intervention in TRS. While rTMS is often conceptualised as a circuit-targeted intervention, heterogeneity in stimulation targets (e.g., prefrontal vs temporoparietal regions) and protocols may have limited its effectiveness in this population. It is also possible that more precise targeting, individualised protocols, or integration with other treatments may be required to achieve clinically meaningful benefits (37–39).

Importantly, this review differs from prior meta-analyses by focusing on treatment-resistant schizophrenia and related populations with persistent refractory symptoms, while applying stricter methodological handling of multi-arm trials, outcome standardisation, and cautious interpretation of heterogeneity and subgroup findings. Unlike previous reviews, the present review explicitly evaluated and reported the certainty of treatment-resistance classification for each study, allowing readers to distinguish findings derived from clearly defined TRS populations from those derived from studies in which treatment resistance was incompletely reported. These differences may explain discrepancies with earlier findings and provide a more transparent and clinically relevant estimate of rTMS effects in this population.

This review has several strengths. It focuses exclusively on TRS, a clinically important but under-represented subgroup, and includes only randomized controlled trials, enhancing internal validity. The statistical approach was robust, using REML-based random-effects models with reporting of heterogeneity and prediction intervals, allowing a more nuanced interpretation of uncertainty.

However, several limitations must be considered. The number of included studies remains relatively small, particularly for outcomes such as auditory hallucinations, limiting statistical power. Considerable heterogeneity across studies in terms of stimulation protocols, patient characteristics, and outcome measures further complicates interpretation. Concomitant pharmacological treatments, including ongoing antipsychotic or clozapine use, were not consistently controlled across studies and may have confounded observed treatment effects. Assessment of publication bias was limited by the small number of studies available for each outcome. Consequently, publication bias was explored descriptively through visual inspection of funnel plots rather than formal asymmetry tests (**Supplementary Figures 5-7**) The possibility of publication bias or small-study effects therefore cannot be excluded. In addition, this review included only English-language peer-reviewed publications and did not systematically search grey literature sources, which may have introduced language or publication bias. Furthermore, studies published after the final search date (10^th^ September 2025) were not captured.

Moreover, most studies reported only short-term outcomes, with limited data on long-term efficacy, functional outcomes, or quality of life. Although adverse events and treatment discontinuation were examined qualitatively, safety reporting varied considerably across studies and often lacked standardized definitions or detailed event counts, precluding quantitative synthesis of safety outcomes.

A key limitation relates to the definition of treatment resistance across included studies. Although this review focused on treatment-resistant schizophrenia, only a subset of studies explicitly defined TRS using established criteria such as failure to respond to at least two adequate antipsychotic trials or clozapine-resistant illness. Several studies enrolled participants with persistent auditory hallucinations, predominant negative symptoms, or chronic schizophrenia without reporting formal TRS criteria. Consequently, some included populations may not fully correspond to contemporary operational definitions of TRS. These limitations reflect the reporting practices of the historical rTMS literature and should be considered when interpreting the findings. Future studies should adopt standardized consensus definitions of treatment resistance to improve comparability across trials.

An additional methodological limitation is that effect sizes were calculated using post-treatment outcome scores rather than change scores. Although this approach is accepted for randomized controlled trials because randomization is expected to minimize systematic baseline differences between treatment groups, residual baseline imbalances may influence effect estimates, particularly in smaller studies. Baseline symptom scores for the active and sham groups were extracted and are presented in the Supplementary Material (**Supplementary Table 2**). Overall, these values appeared broadly comparable across treatment arms, although some individual studies showed modest differences that may reflect the small sample sizes. Therefore, while the assumption of baseline comparability is generally supported, it cannot be completely verified for all included studies.

Low statistical heterogeneity (I²) observed in some analyses should be interpreted cautiously, as such estimates may be unreliable when based on a small number of studies and do not necessarily reflect true clinical consistency, particularly in the presence of substantial variability in stimulation protocols. These limitations highlight important directions for future research. Larger, well-powered trials with standardised protocols are needed to clarify whether specific stimulation parameters or patient subgroups may benefit from rTMS. Future studies should incorporate longer follow-up periods and include patient-centred outcomes to better assess clinical relevance. There is also a need for more precise, hypothesis-driven targeting approaches, as well as evaluation of rTMS as part of multimodal treatment strategies rather than a standalone intervention.

One important limitation relates to the scope of positive symptom assessment. Although the review aimed to evaluate the effects of rTMS on global symptom severity, negative symptoms, and auditory hallucinations in treatment-resistant schizophrenia, assessment of the broader positive symptom domain was largely limited to auditory hallucinations measured using the AHRS. PANSS total scores were included as a measure of global symptom severity rather than a positive-symptom-specific outcome. Other important positive symptom domains, including delusions, formal thought disorder, and disorganized thinking, were not separately evaluated due to limited and inconsistent reporting across studies. Therefore, the present findings should not be interpreted as evidence regarding the broader positive symptom domain as a whole, but rather primarily reflect the effects of rTMS on auditory hallucinations and overall symptom severity.

An additional limitation is the potential overlap between negative symptoms of schizophrenia and depressive symptoms. Although no statistically significant effects were observed for negative symptoms in this analysis, many included studies did not consistently assess or control for co-morbid depression. As a result, it remains difficult to determine whether negative symptom measures accurately reflect primary negative symptoms or are influenced by secondary factors such as depressive symptoms. Future studies should incorporate validated measures to distinguish primary negative symptoms from secondary causes, including depression, to improve interpretability of treatment effects. From a clinical perspective, the current evidence does not support routine use of rTMS for symptom reduction in treatment-resistant schizophrenia. While rTMS appears generally well tolerated, larger and methodologically rigorous trials are needed before it can be recommended as a standard adjunctive treatment in this population.

In summary, current evidence does not support a consistent therapeutic benefit of rTMS over sham stimulation for symptom reduction in TRS. While isolated signals of benefit may exist under specific conditions, these findings are not robust and should be interpreted cautiously. Further high-quality, longitudinal, and mechanistically informed trials are required to determine whether rTMS can play a meaningful role in the management of TRS.

## Data Availability

The raw data supporting the conclusions of this article will be made available by the authors, without undue reservation.
