# Supplementary material for: Efficacy and safety of rTMS for global symptom severity, negative symptoms, and auditory hallucinations in treatment-resistant schizophrenia: a systematic review and meta-analysis
Source: Front Psychiatry. 2026 Jul 24;17:1783758. doi: 10.3389/fpsyt.2026.1783758 (PMC13448548; doi:10.3389/fpsyt.2026.1783758)
Supplement: Supplementary file 1 [file SupplementaryFile1.docx]

**SUPPLEMENTARY TABLES**

**Table S1.** Detailed search strategy, restrictions, access dates, and results by each database for the systematic review.

| **Database** | **Restrictions** | **Access date** | **Search strategy** | **No. of results** |
| --- | --- | --- | --- | --- |
| **OVID Embase** | English language, Human | 10 September 2025 | ((Schizophrenia/ OR Psychotic Disorders/ OR (schizophrenia OR psychosis OR psychotic).ti,ab. OR ("treatment resistant schizophrenia" OR "treatment-refractory schizophrenia" OR "refractory schizophrenia" OR TRS).ti,ab.)  AND  (Transcranial Magnetic Stimulation/ OR Neuromodulation/ OR (transcranial magnetic stimulation OR repetitive transcranial magnetic stimulation OR rTMS OR TMS OR neuromodulation).ti,ab. OR ("theta burst stimulation" OR "intermittent theta burst stimulation" OR iTBS OR TBS).ti,ab.))  AND  (randomized controlled trial.pt. OR controlled clinical trial.pt. OR random*.ti,ab. OR placebo*.ti,ab. OR trial*.ti,ab. OR sham.ti,ab.)  limit to (humans and English language) | 1120 |
| **OVID MEDLINE** | English language, Human RCTs | 10 September 2025 | ((Schizophrenia/ OR Psychotic Disorders/ OR (schizophrenia OR psychosis OR psychotic).ti,ab. OR ("treatment resistant schizophrenia" OR "treatment-refractory schizophrenia" OR "refractory schizophrenia" OR TRS).ti,ab.)  AND  (Transcranial Magnetic Stimulation/ OR Neuromodulation/ OR (transcranial magnetic stimulation OR repetitive transcranial magnetic stimulation OR rTMS OR TMS OR neuromodulation).ti,ab. OR ("theta burst stimulation" OR "intermittent theta burst stimulation" OR iTBS OR TBS).ti,ab.))  AND  (randomized controlled trial.pt. OR controlled clinical trial.pt. OR random*.ti,ab. OR placebo*.ti,ab. OR trial*.ti,ab. OR sham.ti,ab.)  limit to (humans and English language) | 278 |
| **OVID PsycINFO** | English language, Human RCTs | 10 September 2025 | ((Schizophrenia/ OR Psychotic Disorders/ OR (schizophrenia OR psychosis OR psychotic).ti,ab. OR ("treatment resistant schizophrenia" OR "treatment-refractory schizophrenia" OR "refractory schizophrenia" OR TRS).ti,ab.)  AND  (Transcranial Magnetic Stimulation/ OR Neuromodulation/ OR (transcranial magnetic stimulation OR repetitive transcranial magnetic stimulation OR rTMS OR TMS OR neuromodulation).ti,ab. OR ("theta burst stimulation" OR "intermittent theta burst stimulation" OR iTBS OR TBS).ti,ab.))  AND  (randomized controlled trial.pt. OR controlled clinical trial.pt. OR random*.ti,ab. OR placebo*.ti,ab. OR trial*.ti,ab. OR sham.ti,ab.)  limit to (humans and English language) | 255 |
| **Scopus** | English language, Human, Article | 10 September 2025 | (TITLE-ABS-KEY(schizophrenia OR psychosis OR psychotic OR "treatment resistant schizophrenia" OR "treatment-refractory schizophrenia" OR "refractory schizophrenia" OR TRS))  AND  (TITLE-ABS-KEY("transcranial magnetic stimulation" OR "repetitive transcranial magnetic stimulation" OR rTMS OR TMS OR neuromodulation OR "theta burst stimulation" OR "intermittent theta burst stimulation" OR iTBS OR TBS))  AND  (TITLE-ABS-KEY(random* OR placebo* OR sham OR trial OR "controlled clinical trial" OR "randomized controlled trial"))  AND (LIMIT-TO(LANGUAGE, "English"))  AND (LIMIT-TO(DOCTYPE, "ar")) | 551 |
| **Web of Science** | English language, Article | 10 September 2025 | TS=(schizophrenia OR psychosis OR psychotic OR "treatment resistant schizophrenia" OR "treatment-refractory schizophrenia" OR "refractory schizophrenia" OR TRS)  AND  TS=("transcranial magnetic stimulation" OR "repetitive transcranial magnetic stimulation" OR rTMS OR TMS OR neuromodulation OR "theta burst stimulation" OR "intermittent theta burst stimulation" OR iTBS OR TBS)  AND  TS=(random* OR placebo* OR sham OR trial OR "controlled clinical trial" OR "randomized controlled trial")  Refined by: DOCUMENT TYPES: (Article) AND LANGUAGES: (English) | 421 |
| **Cochrane** | English language, Article | 10 September 2025 | (schizophrenia:ti,ab,kw OR psychosis:ti,ab,kw OR psychotic:ti,ab,kw OR "treatment resistant schizophrenia":ti,ab,kw OR "treatment-refractory schizophrenia":ti,ab,kw OR "refractory schizophrenia":ti,ab,kw OR TRS:ti,ab,kw)  AND  ("transcranial magnetic stimulation":ti,ab,kw OR "repetitive transcranial magnetic stimulation":ti,ab,kw OR rTMS:ti,ab,kw OR TMS:ti,ab,kw OR neuromodulation:ti,ab,kw OR "theta burst stimulation":ti,ab,kw OR "intermittent theta burst stimulation":ti,ab,kw OR iTBS:ti,ab,kw OR TBS:ti,ab,kw)  AND  (random*:ti,ab,kw OR trial:ti,ab,kw OR placebo*:ti,ab,kw OR sham:ti,ab,kw OR "controlled clinical trial":ti,ab,kw OR "randomized controlled trial":ti,ab,kw) | 610 |
| **ClinicalTrials.gov** | English language, Human | 10 September 2025 | (schizophrenia OR psychosis OR "treatment resistant schizophrenia" OR "refractory schizophrenia" OR TRS) AND ("transcranial magnetic stimulation" OR "repetitive transcranial magnetic stimulation" OR rTMS OR TMS OR neuromodulation OR "theta burst stimulation" OR "intermittent theta burst stimulation" OR iTBS OR TBS) | 267 |
| **PubMed** | English language, Human RCTs | 10 September 2025 | ((("Schizophrenia"[Mesh] OR "Psychotic Disorders"[Mesh])  OR (schizophrenia[tiab] OR psychosis[tiab] OR psychotic[tiab])  OR ("treatment resistant schizophrenia"[tiab] OR "treatment-refractory schizophrenia"[tiab] OR "refractory schizophrenia"[tiab] OR TRS[tiab]))  AND  (("Transcranial Magnetic Stimulation"[Mesh] OR "Neuromodulation"[Mesh])  OR ("transcranial magnetic stimulation"[tiab] OR "repetitive transcranial magnetic stimulation"[tiab] OR rTMS[tiab] OR TMS[tiab] OR neuromodulation[tiab])  OR ("theta burst stimulation"[tiab] OR "intermittent theta burst stimulation"[tiab] OR iTBS[tiab] OR TBS[tiab])))  AND  (randomized controlled trial[pt] OR controlled clinical trial[pt] OR random*[tiab] OR placebo*[tiab] OR trial[tiab] OR sham[tiab]))  AND (humans[Mesh])  AND (english[lang]) | 275 |
| **Total** | | | | 3777 |

**Table S2: Baseline symptom scores for active and sham groups across included studies.**

| **Study ID** | **Active n** | **Active Baseline Mean** | **Active Baseline SD** | **Sham n** | **Sham Baseline Mean** | **Sham Baseline SD** |
| --- | --- | --- | --- | --- | --- | --- |
| **AHRS Total** |  |  |  |  |  |  |
| Bais (2014) | 31 | 26.9 | 6.2 | 16 | 24.8 | 6.0 |
| Blumberger (2012) | 34 | 28.8 | 6.2 | 17 | 26.5 | 7.8 |
| de Jesus (2011) | 8 | 31.0 | 3.6 | 9 | 26.8 | 7.2 |
| Kimura (2016) | 16 | 22.9 | 6.8 | 14 | 24.2 | 7.4 |
| Koops (2016) | 37 | 24.0 | 5.0 | 34 | 24.0 | 5.0 |
| Paillere (2017) | 15 | 28.6 | 5.4 | 12 | 28.5 | 5.1 |
| Slotema (2011) | 42 | 26.3 | 6.4 | 20 | 27.4 | 6.9 |
| **PANSS Total** |  |  |  |  |  |  |
| Bais (2014) | 31 | 28.9 | 7.6 | 16 | 32.5 | 9.4 |
| Blumberger (2012) | 34 | 68.3 | 17.9 | 17 | 61.9 | 14.2 |
| Chauhan (2021) | 19 | 93.2 | 9.4 | 17 | 90.6 | 8.1 |
| Du (2022) | 25 | 72.1 | 15.3 | 22 | 69.3 | 11.5 |
| Fitzgerald (2008) | 12 | 67.3 | 11.7 | 8 | 60.5 | 11.8 |
| Li (2025) | 60 | 73.3 | 12.3 | 30 | 69.4 | 11.8 |
| Prikryl (2007) | 11 | 57.7 | 8.4 | 11 | 64.0 | 13.1 |
| Wang (2022) | 33 | 71.1 | 18.8 | 26 | 71.5 | 18.8 |
| **Negative Symptoms** |  |  |  |  |  |  |
| Bais (2014) | 31 | 14.4 | 4.7 | 16 | 16.6 | 5.6 |
| Basavaraju (2021) | 15 | 85.2 | 11.8 | 15 | 82.5 | 13.8 |
| Chauhan (2021) | 19 | 27.3 | 3.4 | 17 | 26.5 | 3.1 |
| Du (2022) | 25 | 88.1 | 17.9 | 22 | 88.1 | 15.2 |
| Fitzgerald (2008) | 12 | 55.5 | 4.1 | 8 | 60.5 | 10.3 |
| Li (2025) | 60 | 26.2 | 5.8 | 30 | 24.0 | 5.2 |
| Paillere (2017) | 15 | 40.7 | 18.0 | 12 | 43.1 | 33.6 |
| Prikryl (2007) | 11 | 64.3 | 13.5 | 11 | 59.6 | 19.1 |
| Singh (2020) | 15 | 59.1 | 14.7 | 15 | 70.0 | 15.1 |
| Wang (2022) | 33 | 38.6 | 15.3 | 26 | 39.2 | 9.5 |

**Table S3. Outcome-level risk of bias assessment of included randomized controlled trials using the Cochrane RoB 2 tool.**

| **Study** | **Randomization** | **Deviations** | **Missing Data** | **Measurement** | **Reporting** | **Overall** |
| --- | --- | --- | --- | --- | --- | --- |
| Bais (2014) | Low | Low | Low | Low | Low | Low |
| Basavaraju (2021) | Low | Low | Low | Low | Low | Low |
| Blumberger (2012) | Some concerns | Some concerns | Some concerns | Low | Low | Some concerns |
| Chauhan (2021) | Low | Low | Low | Low | Low | Low |
| de Jesus (2011) | Low | Low | Low | Low | Low | Low |
| Du (2022) | Low | Low | Some concerns | Low | Low | Some concerns |
| Fitzgerald (2008) | Low | Low | Some concerns | Low | Low | Some concerns |
| Kimura (2016) | Low | Low | Low | Low | Low | Low |
| Koops (2016) | Low | Low | Some concerns | Low | Low | Some concerns |
| Li (2025) | Low | Low | Some concerns | Low | Low | Some concerns |
| Paillère-Martinot (2017) | Low | Low | Low | Low | Low | Low |
| Prikryl (2007) | Low | Low | Low | Low | Low | Low |
| Singh (2020) | Low | Low | Some concerns | Low | Low | Some concerns |
| Slotema (2011) | Low | Low | High | Low | Low | High |
| Wang (2022) | Low | Low | Low | Low | Low | Low |

### **Subgroup Analysis**

**Table S4:** Subgroup analyses of the standardized mean difference between active rTMS and sham stimulation for auditory hallucinations measured using the Auditory Hallucination Rating Scale (AHRS).

| **Subgroup** | **k** | **Sample Size** | **SMD (95% CI Lower-Upper)** | **I^2^** |
| --- | --- | --- | --- | --- |
| AHRS | 7 | 305 | ˗0.03 (˗0.26–0.20) | 0% |
| **Country** |  |  |  |  |
| Netherlands | 3 | 180 | ˗0.04 (˗0.34–0.27) | 4% |
| France | 1 | 27 | ˗0.29 (˗1.06–0.47) | NA |
| Brazil | 1 | 17 | ˗0.09 (˗1.04 –0.87) | NA |
| Canada | 1 | 51 | ˗0.08 (˗0.67–0.50) | NA |
| Japan | 1 | 30 | 0.33 (˗0.39–1.06) | NA |
| **Settings** |  |  |  |  |
| Outpatients | 4 | 211 | ˗0.01 (˗0.28–0.27) | 0% |
| Mixed | 1 | 47 | ˗0.40 (˗1.01–0.21) | NA |
| NR | 2 | 47 | 0.18 (˗0.40–0.76) | 0% |
| **TRS Definition** |  |  |  |  |
| ≥2 failed AP trials | 6 | 234 | ˗0.04 (˗0.31–0.23) | 0% |
| Frequent AVH | 1 | 71 | 0.00 (˗0.47–0.47) | NA |
| **Stimulation Site** |  |  |  |  |
| Temporoparietal | 6 | 243 | ˗0.09 (˗0.34–0.17) | 0% |
| Temporal | 1 | 62 | 0.20 (˗0.34–0.73) | NA |

**Table S5:** Subgroup analyses of the standardized mean difference between active rTMS and sham stimulation for global symptom severity measured using the Positive and Negative Syndrome Scale (PANSS) total score

| **Subgroup** | **k** | **Sample Size** | **SMD (95% CI Lower-Upper)** | **I^2^** |
| --- | --- | --- | --- | --- |
| PANSS | 8 | 372 | 0.14 (˗0.26–0.54) | 68% |
| **Country** |  |  |  |  |
| China | 3 | 196 | 0.24 (˗0.39–0.88) | 77% |
| Australia | 1 | 20 | ˗0.68 (˗1.61–0.24) | NA |
| India | 1 | 36 | ˗0.41 (˗1.07–0.25) | NA |
| Canada | 1 | 51 | ˗0.16 (˗0.74–0.42) | NA |
| Netherlands | 1 | 47 | 0.52 (˗0.10–1.13) | NA |
| Czech Republic | 1 | 22 | 1.14 (0.23–2.06) | NA |
| **Settings** |  |  |  |  |
| Inpatients | 4 | 195 | 0.07 (˗0.48–0.62) | 62% |
| Outpatients | 3 | 130 | 0.07 (˗0.83–0.97) | 82% |
| Mixed | 1 | 47 | 0.52 (˗0.10–1.13) | NA |
| **TRS Definition** |  |  |  |  |
| ≥2 failed AP trials | 5 | 213 | 0.08 (˗0.49–0.65) | 74% |
| Significant negative symptoms | 2 | 69 | 0.42 (˗0.90–1.74) | 83% |
| Unclear | 1 | 90 | 0.05 (˗0.39–0.49) | NA |
| **Stimulation Site** |  |  |  |  |
| Prefrontal | 5 | 238 | 0.24 (˗0.37–0.85) | 75% |
| Temporoparietal | 2 | 98 | 0.17 (˗0.49–0.83) | 59% |
| Cerebellar Vermis | 1 | 36 | ˗0.41 (˗1.07–0.25) | NA |

Subgroup evaluations of PANSS score across geographic regions, clinical settings, and definitions of TRS largely mirrored this primary finding, with most results remaining non-significant. While the Czech Republic reported a significant individual study effect (SMD = 1.14; 95% CI: 0.23 to 2.06), pooled results from other nations and various stimulation sites, including prefrontal, temporoparietal, and cerebellar vermis, failed to reach statistical significance. Furthermore, high heterogeneity persisted across inpatient (I^2^ = 62%) and outpatient (I^2^ = 82%) settings, suggesting that variations in rTMS protocols or patient populations may obscure a consistent therapeutic signal in global symptom severity.

**Table S6:** Subgroup analyses of the standardized mean difference between active rTMS and sham stimulation for negative symptoms measured using the Scale for the Assessment of Negative Symptoms (SANS) or the Positive and Negative Syndrome Scale (PANSS) negative subscale.

| **Subgroup** | **k** | **Sample Size** | **SMD (95% CI Lower-Upper)** | **I^2^** |
| --- | --- | --- | --- | --- |
| SANS/PANSS Negative | 10 | 408 | 0.37 (˗0.12–0.86) | 77% |
| **Country** |  |  |  |  |
| India | 3 | 96 | ˗0.16 (˗1.62–1.29) | 90% |
| China | 3 | 196 | 0.46 (˗0.02–0.94) | 61% |
| France | 1 | 27 | ˗0.01 (˗0.77–0.75) | NA |
| Netherlands | 1 | 47 | 0.52 (˗0.09–1.14) | NA |
| Australia | 1 | 20 | 1.42 (0.40–2.44) | NA |
| Czech Republic | 1 | 22 | 1.07 (0.16–1.97) | NA |
| **Settings** |  |  |  |  |
| Inpatients | 4 | 195 | 0.23 (˗0.13–0.58) | 44% |
| Outpatients | 4 | 136 | 0.86 (0.27–1.45) | 57% |
| Mixed | 2 | 77 | ˗0.43 (˗2.33–1.47) | 93% |
| **TRS Definition** |  |  |  |  |
| ≥2 failed AP trials | 6 | 219 | 0.20 (˗0.58– 0.97) | 84% |
| Significant negative symptoms | 3 | 99 | 0.76 (0.11–1.41) | 57% |
| Unclear | 1 | 90 | 0.22 (˗0.22–0.66) | NA |
| **Stimulation Site** |  |  |  |  |
| Prefrontal | 6 | 268 | 0.75 (0.33–1.17) | 58% |
| Cerebellar Vermis | 2 | 66 | ˗0.81 (˗1.95–0.33) | 79% |
| Temporoparietal | 2 | 74 | 0.30 (˗0.21–0.82) | 13% |

**SUPPLEMENTARY FIGURES**

### **Sensitivity Analysis**

**Figure S1.** Leave-one-out sensitivity analysis for the effect of active rTMS versus sham stimulation on auditory hallucinations measured using the Auditory Hallucination Rating Scale (AHRS) (k=7).

Using a "leave-one-out" approach, the analysis reveals that the omission of any single study does not significantly shift the pooled estimate for AHRS, with the SMD remaining consistently near the null (ranging from –0.09 to 0.03) and p-values consistently exceeding 0.05. Notably, the heterogeneity remains at 0% (I^2^ = 0%) across all iterations, indicating a high degree of consistency between the included trials. The final random effects model confirms a non-significant difference between active and sham interventions, suggesting that no individual study exerted undue influence on the overall conclusion that the treatment effect is negligible for this outcome.

**Figure S2.** Leave-one-out sensitivity analysis for the effect of active rTMS versus sham stimulation on global psychotic symptom severity measured using the Positive and Negative Syndrome Scale (PANSS) total score (k=8).

**Figure S3.** Leave-one-out sensitivity analysis for the effect of active rTMS versus sham stimulation on negative symptoms measured using the Scale for the Assessment of Negative Symptoms (SANS) or the Positive and Negative Syndrome Scale (PANSS) negative subscale (k=10).

### **Figure S4:** Comparison of pooled standardized mean differences and confidence intervals using conventional Wald-type and Hartung-Knapp random-effects models.

| **Parameter** | **Wald/Standard** | **Hartung-Knapp** |
| --- | --- | --- |
| Overall AHRS |  |  |
| Overall PANSS |  |  |
| Overall negative symptoms (SANS/PANSS negative subscale) |  |  |
| ***Note:*** Hartung-Knapp sensitivity analyses produced the same pooled SMD estimates as the primary Wald analyses and did not materially change the interpretation of the findings. The overall conclusions remained unchanged for AHRS, PANSS total, and negative symptom outcomes. | | |

**Figure S5:**  Funnel plot evaluating small-study effects for auditory hallucination outcomes measured using the Auditory Hallucination Rating Scale (AHRS).

**Figure S6:** Funnel plot evaluating small-study effects for global symptom severity outcomes measured using the Positive and Negative Syndrome Scale (PANSS) total score.

**Figure S7:**  Funnel plot evaluating small-study effects for negative symptom outcomes measured using the Scale for the Assessment of Negative Symptoms (SANS) or the Positive and Negative Syndrome Scale (PANSS) negative subscale.

**REFERENCES**

[1] Bais, L., Vercammen, A., Stewart, R., Van Es, F., Visser, B., Aleman, A., & Knegtering, H. (2014). Short and long term effects of left and bilateral repetitive transcranial magnetic stimulation in schizophrenia patients with auditory verbal hallucinations: a randomized controlled trial. *PloS one, 9*(10), e108828.

[2] Basavaraju, R., Ithal, D., Thanki, M. V., Ramalingaiah, A. H., Thirthalli, J., Reddy, R. P., ... & Kesavan, M. (2021). Intermittent theta burst stimulation of cerebellar vermis enhances fronto-cerebellar resting state functional connectivity in schizophrenia with predominant negative symptoms: a randomized controlled trial. *Schizophrenia research, 238*, 108-120.

[3] Blumberger, D. M., Christensen, B. K., Zipursky, R. B., Moller, B., Chen, R., Fitzgerald, P. B., & Daskalakis, Z. J. (2012). MRI-targeted repetitive transcranial magnetic stimulation of Heschl’s gyrus for refractory auditory hallucinations. *Brain stimulation, 5*(4), 577-585.

[4] Chauhan, P., Garg, S., Tikka, S. K., & Khattri, S. (2021). Efficacy of intensive cerebellar intermittent theta burst stimulation (iCiTBS) in treatment-resistant schizophrenia: a randomized placebo-controlled study. *The Cerebellum, 20*(1), 116-123.

[5] de Jesus, D. R., Gil, A., Barbosa, L., Lobato, M. I., da Silva Magalhães, P. V., de Souza Favalli, G. P., ... & da Silva Belmonte-de-Abreu, P. (2011). A pilot double-blind sham-controlled trial of repetitive transcranial magnetic stimulation for patients with refractory schizophrenia treated with clozapine. *Psychiatry research, 188*(2), 203-207.

[6] Du, X. D., Li, Z., Yuan, N., Yin, M., Zhao, X. L., Lv, X. L., ... & Zhang, X. Y. (2022). Delayed improvements in visual memory task performance among chronic schizophrenia patients after high-frequency repetitive transcranial magnetic stimulation. *World Journal of Psychiatry, 12*(9), 1169.

[7] Fitzgerald, P. B., Herring, S., Hoy, K., McQueen, S., Segrave, R., Kulkarni, J., & Daskalakis, Z. J. (2008). A study of the effectiveness of bilateral transcranial magnetic stimulation in the treatment of the negative symptoms of schizophrenia. *Brain Stimulation, 1*(1), 27-32.

[8] Kim, E. J., Yeo, S., Hwang, I., Park, J. I., Cui, Y., Jin, H. M., ... & Chung, Y. C. (2014). Bilateral repetitive transcranial magnetic stimulation for auditory hallucinations in patients with schizophrenia: a randomized controlled, cross-over study. *Clinical Psychopharmacology and Neuroscience, 12*(3), 222.

[9] Kimura, H., Kanahara, N., Takase, M., Yoshida, T., Watanabe, H., & Iyo, M. (2016). A randomized, sham-controlled study of high frequency rTMS for auditory hallucination in schizophrenia. *Psychiatry research, 241*, 190-194.

[10] Koops, S., Dellen, E. V., Schutte, M. J., Nieuwdorp, W., Neggers, S. F., & Sommer, I. E. (2016). Theta burst transcranial magnetic stimulation for auditory verbal hallucinations: negative findings from a double-blind-randomized trial. *Schizophrenia bulletin, 42*(1), 250-257.

[11] Lee, S. H., Kim, W., Chung, Y. C., Jung, K. H., Bahk, W. M., Jun, T. Y., ... & Chae, J. H. (2005). A double blind study showing that two weeks of daily repetitive TMS over the left or right temporoparietal cortex reduces symptoms in patients with schizophrenia who are having treatment-refractory auditory hallucinations. *Neuroscience letters, 37*6(3), 177-181.

[12] Li, J., Lu, X., Du, S., Hu, W., Xiao, D., He, B., ... & Li, Z. (2025). Effects of Varied rTMS Frequencies on Cognitive Function in Individuals with Chronic Schizophrenia: A Double-Blind Randomized Controlled Trial. *Journal of Psychiatric Research.*

[13] Loo, C. K., Sainsbury, K., Mitchell, P., Hadzi-Pavlovic, D., & Sachdev, P. S. (2010). A sham-controlled trial of left and right temporal rTMS for the treatment of auditory hallucinations. *Psychological medicine, 40*(4), 541-546.

[14] McIntosh, A. M., Semple, D., Tasker, K., Harrison, L. K., Owens, D. G., Johnstone, E. C., & Ebmeier, K. P. (2004). Transcranial magnetic stimulation for auditory hallucinations in schizophrenia. *Psychiatry research, 127*(1-2), 9-17.

[15] Paillère‐Martinot, M. L., Galinowski, A., Plaze, M., Andoh, J., Bartrés‐Faz, D., Bellivier, F., ... & Artiges, E. (2017). Active and placebo transcranial magnetic stimulation effects on external and internal auditory hallucinations of schizophrenia. *Acta Psychiatrica Scandinavica, 135*(3), 228-238.

[16] Prikryl, R., Kasparek, T., Skotakova, S., Ustohal, L., Kucerova, H., & Ceskova, E. (2007). Treatment of negative symptoms of schizophrenia using repetitive transcranial magnetic stimulation in a double-blind, randomized controlled study. *Schizophrenia research, 95*(1-3), 151-157.

[17] Singh, S., Kumar, N., Verma, R., & Nehra, A. (2020). The safety and efficacy of adjunctive 20-Hz repetitive transcranial magnetic stimulation for treatment of negative symptoms in patients with schizophrenia: a double-blinded, randomized, sham-controlled study. Indian *Journal of Psychiatry, 62*(1), 21-29.

[18] Slotema, C. W., Blom, J. D., de Weijer, A. D., Diederen, K. M., Goekoop, R., Looijestijn, J., ... & Sommer, I. E. (2011). Can low-frequency repetitive transcranial magnetic stimulation really relieve medication-resistant auditory verbal hallucinations? Negative results from a large randomized controlled trial. *Biological psychiatry, 69*(5), 450-456.

[19] Wang, L., Li, Q., Wu, Y., Ji, G. J., Wu, X., Xiao, G., ... & Wang, K. (2022). Intermittent theta burst stimulation improved visual-spatial working memory in treatment-resistant schizophrenia: A pilot study. *Journal of Psychiatric Research, 149*, 44-53.

**META-ANALYSIS CODES**

**Overall Meta-Analysis**

meta.main = function(data) {

  base::return(

    meta::metacont(

      n.e = sham_n,

      mean.e = sham_post_mean,

      sd.e = sham_post_sd,

      n.c = active_n,

      mean.c = active_post_mean,

      sd.c = active_post_sd,

      studlab = Study_ID,

      data = data,

      sm = "SMD",

      method.smd = "Hedges",

      method.tau = "REML",

      method.random.ci = "classic",

      prediction = TRUE,

      random = TRUE,

      common = FALSE

    )

  )

}

**Subgroup Analysis**

meta.subgroup = function(data, value) {

  base::return(

    meta::metacont(

      n.e = sham_n,

      mean.e = sham_post_mean,

      sd.e = sham_post_sd,

      n.c = active_n,

      mean.c = active_post_mean,

      sd.c = active_post_sd,

      studlab = Study_ID,

      data = data,

      sm = "SMD",

      method.smd = "Hedges",

      method.tau = "REML",

      method.random.ci = "classic",

      prediction = TRUE,

      random = TRUE,

      common = FALSE,

      subgroup = data[[value]],

      subgroup.name = value

    )

  )

}

**LOO Analysis**

loo.main = function(data) {

  curr.loo = meta::metacont(

    n.e = sham_n,

    mean.e = sham_post_mean,

    sd.e = sham_post_sd,

    n.c = active_n,

    mean.c = active_post_mean,

    sd.c = active_post_sd,

    studlab = Study_ID,

    data = data,

    sm = "SMD",

    method.smd = "Hedges",

    method.tau = "REML",

    method.random.ci = "classic",

    random = TRUE,

    common = FALSE

  )

  base::return(

    meta::metainf(

      curr.loo,

      pooled = "random"

    )

  )

}

**Publication Bias Funnel Plot**

funnel.main = function(meta) {

  meta::funnel(

    meta,

    xlab = "SMD",

    ylab = "Standard Error",

    pch = 19,

    col = "blue",

    cex = 1.2,

    contour.levels = c(0.90, 0.95, 0.99),

    col.contour = c("gray70", "gray60", "gray50"),

    col.diagonal = "black",

    backtrans = FALSE

  )

  graphics::legend(

    "topright",

    legend = c("p > 0.10", "0.05 < p < 0.10", "0.01 < p < 0.05", "p < 0.01"),

    fill = c("gray90", "gray70", "gray60", "gray50"),

    cex = 0.8

  )

}
